# Embryonic exposure to the fungicide vinclozolin causes virilization of females and alteration of progesterone receptor expression *in vivo*: an experimental study in mice

**DOI:** 10.1186/1476-069X-5-4

**Published:** 2006-02-21

**Authors:** Jill Buckley, Emily Willingham, Koray Agras, Laurence S Baskin

**Affiliations:** 1 Institute for the Study and Treatment of Hypospadias, Department of Urology, School of Medicine, University of California, San Francisco, Parnassus Campus, HSW 1434, San Francisco, CA 94143, USA

## Abstract

**Background:**

Vinclozolin is a fungicide that has been reported to have anti-androgenic effects in rats. We have found that *in utero *exposure to natural or synthetic progesterones can induce hypospadias in mice, and that the synthetic progesterone medroxyprogesterone acetate (MPA) feminizes male and virilizes female genital tubercles. In the current work, we selected a relatively low dose of vinclozolin to examine its *in utero *effects on the development of the genital tubercle, both at the morphological and molecular levels.

**Methods:**

We gave pregnant dams vinclozolin by oral gavage from gestational days 13 through 17. We assessed the fetal genital tubercles from exposed fetuses at E19 to determine location of the urethral opening. After determination of gonadal sex, either genital tubercles were harvested for mRNA quantitation, or urethras were injected with a plastic resin for casting. We analyzed quantified mRNA levels between treated and untreated animals for mRNA levels of estrogen receptors α and β, progesterone receptor, and androgen receptor using nonparametric tests or ANOVA. To determine effects on urethral length (males have long urethras compared to females), we measured the lengths of the casts and performed ANOVA analysis on these data.

**Results:**

Our morphological results indicated that vinclozolin has morphological effects similar to those of MPA, feminizing males (hypospadias) and masculinizing females (longer urethras). Because these results reflected our MPA results, we investigated the effects of *in utero *vinclozolin exposure on the mRNA expression levels of androgen, estrogen α and β, and progesterone receptors. At the molecular level, vinclozolin down-regulated estrogen receptor α mRNA in females and up-regulated progesterone receptor mRNA. Vinclozolin-exposed males exhibited up-regulated estrogen receptor α and progesterone receptor mRNA, effects we have also seen with exposure to the synthetic estrogen, ethinyl estradiol.

**Conclusion:**

The results suggest that vinclozolin virilizes females and directly or indirectly affects progesterone receptor expression. It also affects estrogen receptor expression in a sex-based manner. We found no *in vivo *effect of vinclozolin on androgen receptor expression. We propose that vinclozolin, which has been designated an anti-androgen, may also exert its effects by involving additional steroid-signaling pathways.

## Introduction

Vinclozolin has been identified as an anti-androgenic endocrine disruptor that produces malformations related to androgen inhibition in rats [1]. This fungicide, used on food crops (soft fruits and vegetables) such as grapes used in wine [2], can be toxic in high doses, but its "no observed adverse effects level" is considered to be between 1 and 3 mg/kg in rats [3].

Vinclozolin is known to affect androgen-receptor-mediated endpoints [1,4-8], but some research on behaviors inferred to be androgen-mediated indicates that progesterone receptors may also be involved in the manifestation of these behaviors since mice lacking the progesterone receptor exhibit alterations or decreases in these ostensibly androgen-mediated behaviors [9,10]. Vinclozolin has been shown to affect androgen-receptor-mediated behaviors in mammals [11] and birds [12]. In addition, research indicates an inability for vinclozolin to compete for androgen receptor in the fathead minnow [13], even though it disrupts male steroid profiles and alters female gonadal condition in adults [13]. Although most of these studies are behavioral, they do imply the involvement of steroid receptors other than androgen receptors in these processes.

In a study with rats, androgen receptor mRNA was unaffected by vinclozolin exposure [5]. Laws et al. [14] found that the two anti-androgenic metabolites of vinclozolin can compete with progesterone for progesterone receptor *in vitro*, but do not bind estrogen receptor. Their *in vivo *studies, however, indicated that vinclozolin did not disrupt progesterone-receptor-based pathways in adult animals; for example, it elicited no change in ovulation in adult female rats, and the researchers found no changes in progesterone receptor distribution after short-term vinclozolin exposure.

The study by Laws et al. [14] was performed using adult rats. Part of the endocrine-disruptor model is the choice of sensitive developmental time-points at which the organism may be most susceptible to exposure, especially to low-dose exposure (Crews et al. 2000). We investigated how a relatively low dose of vinclozolin affected fetal genital tubercle development in mice, a steroid-dependent process. Our results led us to conduct further investigations into the effects of *in utero *vinclozolin exposure on steroid receptor expression in the genital tubercle, especially progesterone receptor.

## Materials and methods

### Casting

Timed-pregnant CD1 mice were received on gestational day 8 (Charles River Laboratories, Wilmington, MA, USA) and housed in separate animal cages until embryonic stage (E) 13. All animals were housed one per cage (20 × 25 × 47 cm) with laboratory-grade pine shavings (heat-treated to remove resins) as bedding. They were acclimated to 68–74°F and 40%–50% relative humidity on a reversed light schedule (14 hr light and 10 hr dark) and received ad libitum mouse chow and water. All animal-related procedures described here were approved by our institutional animal care and use committee.

The pregnant mice were separated into three groups (N > 6 each); vehicle control (corn oil only), a low dose of vinclozolin (D1 = 10 mg/kg dissolved in corn oil), and a higher dose of vinclozolin (D2 = 50 mg/kg dissolved in corn oil). On gestational day (GD) 13 (the start of genital tubercle differentiation), we began daily gavaging of the pregnant mice with 0.1 ml of either corn oil (control), D1 vinclozolin, or D2 vinclozolin. Gavaging ended on gestational day 17. On GD 19, dams were killed by CO_2 _followed by cervical dislocation, and the fetuses were harvested via cesarean section and sex was determined by microscopic examination of gonads.

For casting, each fetal bladder/urethra was cast by injection (Baston's No. 17 Plastic Replica and Corrosion Kit (Polysciences, Inc., Warrington, PA, USA) using a 30 gauge needle with 3× microscopic visualization. The casting material was injected into the bladder until it emerged from the urethral opening. Casts set overnight at 4°C and were transferred to 20% potassium hydroxide the following day to dissolve the remaining tissue. The fetal bladder/urethra casts were photographed using a digital camera (Nikon 900, Melville, NY, USA). Using Adobe Photoshop 7.0 (Apple, Sunnyvale), we obtained fetal urethral measurements of urethral length based on pixel count. In the male pups, additional measurements were made from the prostate gland to Cowper's gland and from Cowper's gland to the distal urethral tip.

### RT-PCR and macroscopic assessment of hypospadias

Timed pregnant CD1 mice (Charles River Breeding Laboratories, Wilmington, MA) were received on GD 8. Mice were gavaged once daily from GD 13 through 17 with 50 mg/kg vinclozolin or with corn oil alone as the vehicle control. We selected the dose of vinclozolin based on results published by Grey et al.^4 ^showing that this was the lowest dose that elicited frank hypospadias in male rats exposed *in utero*; our goal for this set of experiments was to elicit hypospadias for molecular studies of the pathway. We also selected it based on preliminary investigations of our own indicating that this dose would produce hypospadias at a frequency that would allow us to compare tissue from hypospadic and nonhypospadic animals at the molecular level. All animals were housed as described above. Fetal genital tubercle morphology was assessed and tissue harvested on GD 19 (when urethral closure at the tip of genital tubercle normally has occurred) [16]. Dams were killed by CO_2 _followed by cervical dislocation and fetuses removed. After gross examination and identification of the location of the urethral opening (either low or normal), gonadal sex was identified, and genital tubercles were harvested by microdissection and placed in RNAlater (Qiagen) for RNA isolation for RT-PCR analysis.

#### Visual assessment of urethra

Gross assessment of the location of the urethral opening was made primarily by expressing the bladder and observing the emergence of fluid; in normal E19 males, it emerges at the tip of the genital tubercle; in hypospadias it emerges more proximally.

#### Quantitative RT-PCR analysis

Total RNA from pooled tissues was extracted using a kit (Qiagen). It was reverse-transcribed to cDNA and quantitative RT-PCR analysis using the Taqman assay was performed at UCSF's Cancer Center Comprehensive Genome Analysis Core. Primers for the steroid receptors (the two estrogen receptors, progesterone receptor, and androgen receptor) were obtained commercially (Applied Biosystems, Foster City, CA). The gene expression assay used for progesterone receptor (Mm00435625) did not distinguish between the two isoforms; thus, quantification reflects expression of both PR-A and PR-B. Table 1 provides numbers of dams and pups and number of individuals per pooled sample of tissue analyzed. Data for receptor mRNA levels were normalized to the control gene, GapDH. This gene was chosen because it was shown to be the least variable under these experimental conditions (data not shown).

**Table 1:** Summary of experimental conditions for quantitative PCR

	Pup gender	# E19 GTs examined	# pools obtained	# pools omitted	# samples/pool
Control N = 8 (dams)	Females	46	6	0	7–8
	Males w/hypos	0	-	-	-
	Males w/o	48	10	0	4–5
Vinc-treated N = 12 (dams)	Females	49	7	0	7
	Males w/hypos	8	3	0	2–3
	Males w/o	51	6	0	8–9

GT = genital tubercle; hypos = hypospadias.

### Statistical analyses

Statistical analyses were performed using JMP for PC. We used ANOVA analysis on the urethral cast length measurements. RT-PCR data, when not normally distributed, were analyzed and are reported based on a nonparametric median test; we also performed Grubb's outlier tests on RT-PCR data. For normally distributed RT-PCR data, we used ANOVA analysis.

## Results

### Urethral casting

**Figure 1:**
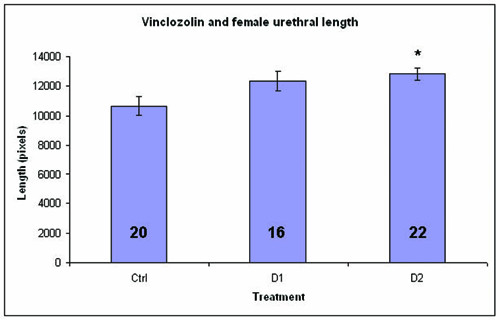
**Length of female urethral casts**. Female urethras were significantly longer in the fetuses from the higher-dose (D2, 50 mg/kg) vinclozolin group. * = P < 0.05 vs. control. A longer urethra indicates virilization of the female. D1 = 10 mg/kg. Ctrl = mice born to dams gavaged with corn oil only.

Urethras from female E19 fetuses exposed to the higher dose of vinclozolin were significantly longer than the control female urethras (P = 0.005) (Fig. 1). In addition, the urethral lengths appeared to exhibit a dose response, with the female urethras from the lower-dose group exhibiting lengths intermediate to the control and higher-dose groups (Fig. 1). Urethras from female exposed to the lower dose of vinclozolin were not significantly longer than those from controls (P = 0.08).

**Figure 2:**
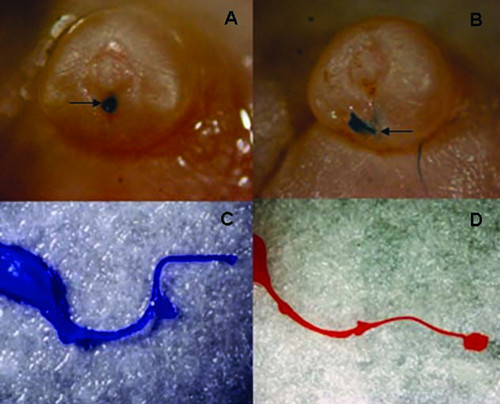
**Male urethras and hypospadias**. Top row shows control (A, left) and treated (B, right, higher-dose group) macroscopic view of male fetal genital tubercle. Note the normal location of the urethral opening (arrow, left) and the more-proximal opening the in treated sample (arrow, right), indicating hypospadias. Urethral openings were visualized by emergence of casting resin from the opening. The bottom row shows a typical control male cast (C, left) and a male urethral cast from the higher-dose group (D, right). Note the lack of an "S" shape and the overall atrophic appearance of the urethra from the treated male (right).

The statistical analysis did not show any differences in urethral length among the males in the groups; however, visually, the urethras from vinclozolin-exposed males appeared considerably less robust and atrophic, and lacking in the typical "S" shape of a control urethra (Fig. 2).

### RT-PCR results

Of the male genital tubercles examined macroscopically, we identified 8 of 59 (13.6%) as having hypospadias. None of the control male genital tubercles were identified as having hypospadias.

#### Progesterone receptor

**Figure 3:**
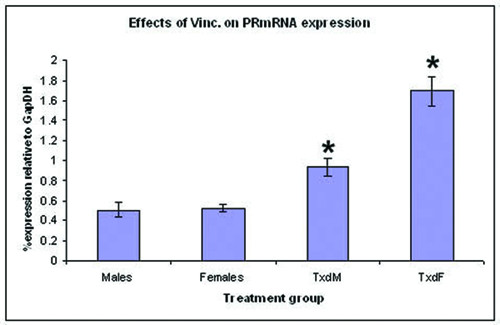
**Progesterone receptor mRNA expression in females and males compared between control and treated groups**. In both sexes, progesterone receptor levels were significantly higher in genital tubercles from treated animals compared to those from control animals. *, P < 0.05 compared to same-sex controls. Males, females = mice born to dams gavaged with corn oil only. TxdM = treated males; TxdF = treated females. Treated animals were born to females gavaged with 50/mg/kg vinclozolin daily from E13 through E17.

Vinclozolin-treated males had higher levels of progesterone receptor mRNA than control males (ANOVA, df = 1; F ratio = 14.1; P = 0.0016) (Fig. 3). In addition, vinclozolin-treated females had significantly higher levels of progesterone receptor mRNA in the genital tubercle (ANOVA, df = 1; F ratio = 52.07; P < 0.0001) (Fig. 3).

When we separated vinclozolin-treated males into two groups, those with and those without hypospadias, we found significant differences between each group compared to controls (P = 0.004 and P = 0.02, respectively) (not shown).

Control males and females did not differ in progesterone receptor mRNA expression (not shown).

#### Estrogen receptors

**Figure 4:**
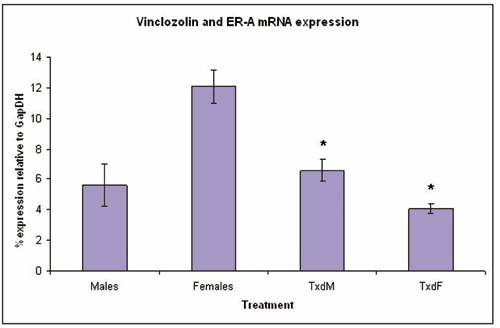
**Estrogen receptor α mRNA expression in females and males compared between control and treated groups**. In vinclozolin-treated females, mRNA expression was significantly lower compared to controls. In treated males, mRNA expression was significantly higher compared to controls. *, P < 0.05 compared to same-sex controls. Males, females = mice born to dams gavaged with corn oil only. TxdM = treated males; TxdF = treated females. Treated animals were born to females gavaged with 50/mg/kg vinclozolin daily from E13 through E17.

Estrogen receptor α was significantly lower in vinclozolin-treated females (ANOVA, df = 1; F ratio = 58.86; P < 0.0001) (Fig. 4). In addition, vinclozolin-treated females exhibited lower levels of estrogen receptor β compared to controls (ANOVA, df = 1; F ratio = 7.62; P = 0.019) (not shown).

We found one outlier among the control males group (Grubb's outlier test) and removed it from the analysis. Estrogen receptor expression α was slightly significantly higher in vinclozolin-treated males than in control males (Wilcoxon's, df = 1; P = 0.03) (Fig. 4).

#### Androgen receptor

We found no significant changes in androgen receptor in comparisons among the groups.

## Discussion

Recent research suggests that compounds we believed were estrogenic may really be disrupting endocrine signaling via the progesterone receptor [17]. Recently some progesterone disruptors have been identified in personal care products [18]. *In utero *exposure to natural or synthetic progesterones can induce hypospadias in mice in which the urethral opening occurs more proximally than normal; the synthetic progesterone medroxyprogesterone acetate (MPA) feminizes male and virilizes female genital tubercles (EW et al. in preparation). In the current work, the relatively low dose of vinclozolin used caused hypospadias in the mice, but it also virilized the females, reflecting our findings with MPA. We also have observed a more proximal urethral opening in females treated with natural progesterone, but have not quantified these data (unpublished data).

Our morphological findings with vinclozolin, in the context of our findings with MPA and progesterone, led us to investigate the effects of vinclozolin on progesterone receptor expression, and on the expression of other steroid hormone receptors. In a study with rats, an *in vitro *assessment of the ability of two vinclozolin metabolites to bind progesterone receptor indicated that it could outcompete the natural ligand; however, the concordant *in vivo *studies from these investigations using adult male rats showed no *in vivo *effect [14]. The discrepancy between these data and our current results could be attributable to species differences or differential responses based on timing of exposure. Our mice experienced vinclozolin exposure *in utero*, rather than as adults, and a central assumption of the endocrine-disruption model is that *in utero *exposures exert different and more powerful effects than do exposures in adults [15]. In addition, our results are consistent between the morphological and molecular levels in males.

Vinclozolin-exposed males exhibited up-regulated estrogen receptor α and up-regulated progesterone receptor, effects we have also seen with exposure to the synthetic estrogen, ethinyl estradiol (unpublished data). Ethinyl estradiol up-regulates estrogen receptor α in females and upregulates progesterone receptor in both sexes. MPA also up-regulates estrogen receptor α and progesterone receptor in males, which it feminizes.

Vinclozolin can cause an increase in aromatase activity [19], which would presumably result in an increase in estradiol, especially in a system where testosterone is being made in abundance, as is the case with the male differentiation pathway. In fact, in the fathead minnow, a species that serves as a model of vertebrate endocrine disruption, vinclozolin exposure resulted in a slight increase in estradiol concentrations in adult male fish [13]. Thus, vinclozolin could act on aromatase, which in turn could act on testosterone, both producing estradiol inappropriately and depriving 5-alpha-reductase of the substrate it needs to produce dihydrotesterone, the androgen necessary for appropriate male development of secondary sex characteristics. One possible explanation for the up-regulation of progesterone receptor mRNA in males could be the boosted estrogen receptor expression; estrogen receptor binds the progesterone receptor promoter. But another potential explanation, given the fact that vinclozolin binds progesterone receptor *in vitro*, is that it is affecting the receptor directly.

At the molecular level, vinclozolin down-regulated expression of estrogen receptors α and β in females and up-regulated progesterone receptor expression. Intuitively, the results with estrogen receptor suggest a lessening effect of estrogen, which would be consistent with our findings of virilization in the females. Androgen receptor expression remained unchanged in females. We speculate that the virilization that occurred in the female genital tubercle may have been the result of indirect estrogen antagonism; vinclozolin does not appear to be an estrogen receptor agonist or antagonist [20]. It may enhance aromatase activity, and the up-regulation in aromatase activity may exhaust available substrate with the ultimate effect of lessening available estrogen. In addition, if signaling through the progesterone receptor is involved in the development of endpoints that are thought to be androgen-mediated, the increase in progesterone receptor in females may provide more opportunity for the development of these ostensibly androgen-associated phenotypes.

## Conclusion

Our findings suggest that vinclozolin directly or indirectly affects progesterone receptor expression and that it also affects estrogen receptor expression in a sex-based manner. We found no *in vivo *effect of vinclozolin on androgen receptor expression in this tissue. We propose that vinclozolin, long designated as an anti-androgen, may also exert its effects by additional steroid-hormone signaling pathways.

## Abbreviations

D1: lower dose of vinclozolin (10 mg/kg)

D2: higher dose of vinclozolin (50 mg/kg)

GD: gestational day

MPA: medroxyprogesterone acetate

## Competing interests

The author(s) declare that they have no competing interests.

## Authors' contributions

JB performed animal treatments, harvested tissue, performed casting, did casting measurements, and wrote portions of the paper. EW collaborated on experimental design, performed animal treatments, harvested tissue, performed the casting, analyzed the data, and wrote portions of the paper. KA treated mice, harvested and collected tissue for quantitative PCR, and performed RNA isolations. LB collaborated on experimental design, participated in data analysis, and wrote portions of the paper.
